# Intra-Capsular Versus Extra-Capsular Breast Mastopexy of Previously Augmented Breast

**DOI:** 10.29252/wjps.7.3.301

**Published:** 2018-09

**Authors:** Hesham A. Helal, Asser El-Hilaly, Nahed Samir Boughdadi

**Affiliations:** Department of Plastic and Reconstructive Surgery Department, Ain Shams University, Cairo, Egypt

**Keywords:** Mastopexy, Breast augmentation, Bottoming out, Bbreast lift, Breast reshaping

## Abstract

**BACKGROUND:**

The augmented breast frequently becomes ptotic by time and most of the patients may seek mastopexy. Although the rate of breast lift surgeries after breast augmentation is increasing, there are few studies regarding the nature of these procedures.

**METHODS:**

Sixty patients with moderate grade ptosis and previously augmented breast by breast implants seeking breast mastopexy. Group A included 30 patients who underwent intra-capsular circum-areolar mastopexy and Group B including another 30 patients who underwent extra-capsular circum-areolar mastopexy. Follow up after complete healing was scheduled at 3, 6, and 12 months post-operative. Frontal and lateral views photography were taken each visit and objective evaluation was carried on by a plastic surgeon not involved in the surgeries. A questionnaire was performed by using the Likert scale to assess patients’ satisfaction.

**RESULTS:**

In group A; the overall rate of complications was 17%, while in group B; the overall rate of complications was 10%. Patients of group A showed overall satisfaction of 4.53±0.69 in comparison to 3.06±0.25 in group B. In group A; objective evaluation, was excellent in 87% while in group B it was excellent in 43%.

**CONCLUSION:**

Reshaping of breast pillars mastopexy augmentation is very important to prevent bottoming-out of the breasts.

## INTRODUCTION

Primary combination of breast augmentation with or without breast lift is a common procedures frequently sought by the patients to enhance the appearance of breasts. Nowadays mastopexy of already augmented breast became almost as popular as the primary procedure due to the increase of poor outcomes of the primary procedure and also increased patients’ demands of a better breast shape.^1^^,^^2^ Unlike the primary one, mastopexy of the augmented breast is more challengeable to the surgeons due to the preexisting scars, stretched thin skin, lack of breast tissues, capsular contraction, breast implant complicationsand higher patients’ expectations.^3^

Two main surgical concepts had been proposed for the mastopexy of the augmented breast to attain the desired result; whether doing mastopexy and plication of the capsule without manipulation of the implant (extra-capsular) or doing the mastopexy procedure with capsular violation and rearranging local breast tissues with or without changing the breast implant (intra-capsular).^4^ Many approaches have been described for the mastopexy. Simple crescent skin excision from the superior pole of the areola; this will only lift the breast few centimeters and so helpful in moderate and large ptosis, in addition neither reshaping of the breast nor change of implant is accessible.^5^

Circum-areolar mastopexy is often useful in secondary cases where it is helpful in mild and moderate ptosis with great flexibility, advantages of this technique is that implant manipulation with proper skin tightening can be achieved without adding more scars.^6^ Disadvantages are widening of the areolar scar and violation of the Nipple Areolar Complex (NAC) vascularity. Conventional mastopexy, using superior, superomedial or superolateral pedicles, is the most efficient means by which both the horizontal and vertical dimensions of the skin brassiere can be reduced, however more scars will be added.^7^ Combination of multiple concepts is mandatory to achieve best aesthetic result, and so we performed this study to evaluate result of two different techniques of mastopexy for patients with previously augmented breast.

## MATERIALS AND METHODS

During the period from January 2013 to April 2017, the study was performed on sixty patients seeking breast reshaping after they had previous breast augmentation for cosmetic purposes. All patients had ptosis of the overlying breast tissue with the implants drooped down in addition to excessive atrophy of the overlying breast tissue. Inclusion criteria were; previous circum-areolar or inframammary sub-glandular breast augmentation with silicone implants, grade 2 or 3 breast ptosisand the position of nipple areola complex ranging from 23-27 cm (measured from the suprasternal notch to the areola).

Exclusion criteria included;evident capsular contracture, ruptured implant, history of diabetes, history of lactation within the year prior to surgery and history of medical breast disorders. An informed consent for the procedure and approval of the study was signed by all patients included. Patients were divided into two groups; Group A: included 30 patients who underwent extra-capsular circum-areolar mastopexyand Group B: including another 30 patients who underwent intra-capsular circum-areolar mastopexy.

Preoperative marks were designed to identify the proper amount of skin excision to tighten overlying skin and suspended breast parenchyma. The breast meridian, inframammary fold location, new NAC position and the periareolar patterns were marked in the standing position. In group A; the areola was incised at a diameter of 4.5- 5 cm, then the outer periareolar pattern was incised and de-epithelialization of the skin in between was done. The dermis was divided in the upper half only between 9 and 3 o’clock leaving a 1 cm dermal rim and dissection was started cranially elevating a superior glandular flap without violation of the implant capsule. Dissection was continued till the level of 2^nd^ rib creating a pocket in the superior pole. After good hemostasis, plication of the capsule was done using a running absorbable monofilament 2-0 suture and fixing it to the pectoral fascia at the 2^nd^ rib level. 

In group B; the areola was incised at a diameter of 4.5-5 cm, then the outer periareolar pattern was incised and de-epithelialization of the skin in between was done. The dermis was divided in the lower pole only from 3-9 o’clock leaving a 1 cm dermal rim. Excision of lower central parenchyma was done and the lower pole of implant capsule was then incised, the implant was removed and the superior border of the capsule was incised and dissection was continued superiorly to allow migration of implant upwards. Next medial and lateral flaps in the lower breast pole were then dissected from the skin to allow breast reshaping inferiorly. Insertion of the new silicone implant then the 2 medial and lateral flaps were sutured using interrupted absorbable monofilament 3-0 sutures and fixed to the pectoralis muscle fascia. 

**Fig. 1:**
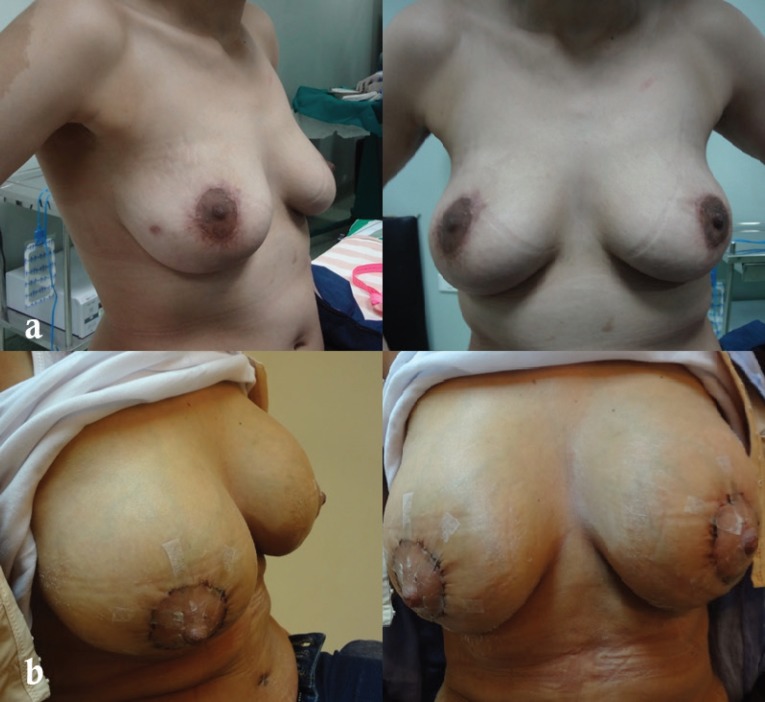
Preoperative (a) and postoperative (b) photos showing a 37 years old patient from group B with previous sub-muscular breast augmentation through circum-areolar incision having intra-capsular mastopexy

**Fig. 2:**
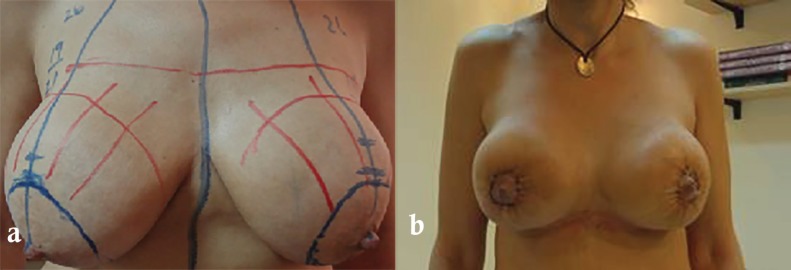
Preoperative (a) and postoperative (b) photos showing a 48 years old patient from group A with previous sub-glandular breast augmentation through infra-mammary incision having extra-capsular mastopexy

For both groups; a 3-0 non-absorbable suture was used to form a purse string suture to fix the areolar size then the areola was closed with interrupted and running 4-0 absorbable monofilament sutures. Follow up after complete healing was scheduled at 3, 6, and 12 months post operatively. Front, lateral and oblique lateral views were taken each visit (Figures 1, 2). A questionnaire was performed to assess patients’ satisfaction covering the five aspects of the result of their surgery; scar, sensation, shape, projection and satisfaction and finally they were asked if they could recommend this operation to her friends or not (Table 1). This questionnaire was performed by using the Likert scale, a psychometric scale commonly used in survey research. Statistical evaluation of differences between the two groups as regarding age, body mass index (BMI), complication rate and sensation was done. 

**Table 1:** Likert Scale; items involved in the questionnaire and their method of evaluation

**Question/Likert item**	**Patient answer**	**Score**
Scar: How do you consider the cosmetic result of the wound?	Extremely poor	1
	Poor	2
	Barely acceptable	3
	Good	4
	Excellent	5
Sensation: Do you have any problem with sensitivity? (for example; numbness, lost sensation, erectile dysfunction)	Always	1
	Often	2
	Sometimes	3
	Rarely	4
	Never	5
Shape: Are u satisfied with the final shape? (size, symmetry, NAC complex, projection, general shape and body harmony)	Extremely poor	1
	Poor	2
	Barely acceptable	3
	Good	4
	Excellent	5
Do you recommend it to your friends?	Strongly disagree	1
	Disagree	2
	Neither agree nor disagree	3
	Agree	4
	Strongly agree	5
Overall satisfaction: How you define your general satisfaction of the surgery?	Extremely poor	1
	Poor	2
	Barely acceptable	3
	Good	4
	Excellent	5

## RESULTS

There was no significant difference between the two groups in age (36±8 versus 38±9 years), mean±SD body mass index was 26±4 versus 27±5. Post-operative follow up period ranged from 12-15 months. Complications were classified into early and late complications (Tables 2 and 3). In group A; the overall rate of complications in group A was 17%, while in group B was 10%. Wound dehiscence was encountered in 3% of cases of each group. In these cases; there was skin dehiscence at the purse-string which was left to heal by secondary intention.

**Table 2:** Early complications in both groups

**Variable**	**Group A**	**Group B**
Wound Dehiscence	1	1
Hematoma	1	0
Wound Infection	0	0
Seroma	0	0
NAC necrosis	0	0
Total	2	1

**Table 3:** Late complications in both groups

**Variable**	**Group A**	**Group B**
Asymmetry (minor)	2	1
Widening of the areola	1	1
Sensation loss	0	0
Total	3	2

Results of the questionnaire performed by all patients were evaluated using the Likert scale. These results showed patients opinion on scars, sensation, final shape and projection. Patients of group B showed obviously superior results as regarding shape and projection that persisted for the first year post-operative. Despite that patients’ opinion on shape and scar was higher 4.56±0.67 in group A, in comparison to 4.1±0.60 in group B, but overall satisfaction was higher in group B 4.2±0.69 (Table 4).

**Table 4:** Results of the questionnaire evaluated by Likert score

**Variable**	**Mean Score±SD ** **(Group A)**	**Mean Score±SD ** **(Group B)**
Scar	4.2±0.97	4.3±0.99
Sensation preserved	4.73±0.56	4.71±0.56
Final shape and projection	3.9±0.67	4.56±0.60
Recommendation	3.3±0.64	4.74±0.67
Overall satisfaction	3.06±0.65	4.57±0.69

Objective evaluation was carried on by a plastic surgeon not involved in the surgeries, through comparison between pre and post-operative photos and by inspection of the final results of breast (volume, shape, symmetry, position of nipple areola complex, longevity of results for one year and cotton test for sensation. Overall results were classified into excellent; good; average and poor showing that in group A, 13, 13, 0 and 4 were excellent, good, average, and poor, respectively; while the figures for group B were 26, 3, 1, and 0, respectively.

## DISCUSSION

In an ideal situation, patients undergoing breast mastopexy surgery desire to have beautifully shaped and positioned breasts without scars. Surgeons can offer corrections for breast size, volume, ptosis and shape but with scars as visible sequelae of the operation. These scars also have psychological impact on patients that require follow up and reassurance.^8^ Secondary mastopexy in the previously augmented patient is an increasingly important topic whose complex surgical and medico-legal implications worth careful attention. As the patients of augmented women get older, many of themusually require combination of breast mastopexy, capsular surgerywith or without implant exchange.^9^

Many methods have been proposed for combined breast mastopexy augmentation varied from just crescent excision from the upper pole of the areola to conventional Wise pattern mastopexy in order to serve combination of breast uplift with reshaping, capsular surgery and implant manipulation. The conventional mastopexy, based on the Wise pattern skin excision has been greatly adopted by surgeons due to its proven versatility as it tightens the skin envelop both vertically and horizontally in addition to the feasibility of internal breast parenchyma suturing, correction of high grades of ptosis, managing breast asymmetry and changing the breast implant if needed. Howeverit usually adds scars to the breast and due to the anatomical and physiological changes in the breast after previous augmentation, excessive dissection is somehow hazardous.^10^

Unlike the previous, circum-areolarmastopexy is usually used only in minimal degrees of ptosis; it is useful in secondary mastopexy due to the limited amount of dissection and thus does not interfere with vascular supply to NAC. Although many techniques were described for reshaping of previously augmented breasts however no single technique has proved superiority over others in lifting the breast and thus the combination of various techniques became mandatory to provide solutions to all arguments faced by surgeons and achieve good final results. In previously augmented patients undergoing secondary mastopexy, there is more reliance on skin resection, flap undermining and dermal adhesion than on parenchymal sutures.^10^

In this study we performed mastopexy in 60 patients who had previously done breast augmentation using the circum-areolar approach with two different modifications for internal reshaping of the breast tissues to maximize the benefit of the technique without addition of more scars. In group A, rearrangement of the breast tissue was done by transfixing the capsule into the pectoral fascia this has the advantages of bringing the implant to a more higher level to add superior fullness, no violation of the capsule, improving the long life of the result, does not engage with the lower pole which is usually the thinnest part of the breast. However, it has some disadvantages in being more complex and does not offer the ability to change the implant if requested by the patient. Also it carries the risk of interference with the vascular supply of the nipple due to extensive dissection.

On the contrary group B patients had mastopexy with capsular tightening and parenchymal rearrangement in the lower pole of the breast and this has the advantages of pushing the implant upwards and forming a strong and stable shelf underneath the implant through the capsular flaps and pillarsto maintain the lifting result for a very long period of time, feasibility of implant exchange as done in all patients, better reshaping of the breast with ability to and also provide coverage of the lower pole of the implant.The disadvantage of this technique is the hazardous dissection of the lower pole.

Although such combined secondary surgery carries increased risks, because of the adverse effects of implants on breast anatomy and physiology in the form of tissue atrophy, thinning and stretching, and reduction of blood supply to the skin and nipple,^11^ we did not report any complication related to vascular compromise due to careful dissection of the parenchymal flaps in a relatively shallow plane to preserve skin blood supply. Hartzell *et al.*,^12^ stated that capsular excision and rearrangement of local tissues in secondary cases can produce an undesired change in implant location. However in our study, we found that excision of a part of the capsule will decrease the space available for the implant and force it up to fill the superior pole of the breast and thus group B patients showed more projection, better shape and longevity of the mastopexy, also this is attributed to the internal suturing of parenchyma and fixing it to the pectoral fascia. 

As in all circum-areolar techniques, we did not address the excess skin in the lower pole in our study, however, most of the patients experiencedhigh overall satisfaction with good breast shape and long term preserved lift without facing unfortunate results. Both extra and intra capsular techniques for mastopexy of previously augmented breast can be used easily for treatment of breast reshaping reduction with satisfactory results. Despite this we thinkthat extracapsular technique is safer, while the intracapsular technique is very attractive to both patient and surgeon due to its good breast contour and shape, upper pole fullness, also longevity of NAC projection and breast contour. In mastopexy augmentation reshaping of breast pillars to support the breast is very important to prevent recurrence.

## CONFLICT OF INTEREST

The authors declare no conflict of interest.
